# Temporal reassignment and correspondence evaluation with quality control for time-course imaging of 3D cell culture

**DOI:** 10.1016/j.crmeth.2025.101237

**Published:** 2025-11-18

**Authors:** Eric M. Cramer, Tamara Lopez-Vidal, Jeanette Johnson, Vania Wang, Daniel R. Bergman, Ashani Weeraratna, Richard Burkhart, Elana J. Fertig, Jacquelyn W. Zimmerman, Laura M. Heiser, Young Hwan Chang

**Affiliations:** 1 Department of Biomedical Engineering, Oregon Health & Science University (OHSU), Portland, OR, USA; 2 Knight Cancer Institute, Oregon Health & Science University, Portland, OR, USA; 3 Department of Oncology, Sidney Kimmel Comprehensive Cancer Center, Johns Hopkins University School of Medicine, Baltimore, MD, USA; 4 Convergence Institute, Johns Hopkins University School of Medicine, Baltimore, MD, USA; 5 Department of Biochemistry and Molecular Biology, Bloomberg School of Public Health, Johns Hopkins University, Baltimore, MD, USA; 6 Institute for Genome Sciences, University of Maryland School of Medicine, Baltimore, MD, USA; 7 Department of Pharmacology and Physiology, University of Maryland School of Medicine, Baltimore, MD, USA; 8 University of Maryland - Institute for Health Computing, University of Maryland School of Medicine, North Bethesda, MD, USA; 9 Department of Surgery, Johns Hopkins University School of Medicine, Baltimore, MD, USA; 10 Bloomberg∼Kimmel Institute for Cancer Immunotherapy, Johns Hopkins University School of Medicine, Baltimore, MD, USA; 11 Department of Medicine, University of Maryland School of Medicine, Baltimore, MD, USA; 12 Greenebaum Comprehensive Cancer Center, University of Maryland School of Medicine, Baltimore, MD, USA

**Keywords:** spheroid, tumor, cancer, organoid, 3D cell culture, extracellular matrix, quality control, imaging, Procrustes

## Abstract

Longitudinal imaging of 3D cell cultures like tumor organoids and spheroids offers crucial insights into cancer progression and treatment. However, spatial displacement during time-course imaging, caused by matrix detachment or experimental artifacts, can confound analyses. We present TRACE-QC, an application of the Procrustes technique to evaluate data integrity and rectify mislabeling in longitudinal imaging of 3D cell culture. Our algorithm integrates permutation-based optimization with Procrustes analysis. By using X and Y coordinates of images, it accurately reorders, matches, and aligns object positions across time points, correcting for global well rotations and translations, along with local spheroid movements. Validation with simulated data confirmed its accuracy and robustness. Applied to longitudinal imaging of tumor spheroids, our algorithm revealed frequent displacement among the spheroids between time points and corrected many mislabeled images. This computationally efficient and adaptable method needs no experimental adjustments and presents a readily accessible solution for data quality control.

## Highlights

• TRACE-QC is a tool that enhances reliability of spheroid-based assays
• Corrects spheroid displacement in 3D cell cultures based on image metadata
• Ensures data integrity for longitudinal imaging

## Motivation

Three-dimensional (3D) *in vitro* models, such as tumor organoids and spheroids embedded in an extracellular matrix, are increasingly vital for studying normal and disease biology, including drug responses. A key advantage of these models is that imaging platforms can perform continuous longitudinal imaging to follow phenotypic changes. However, common issues in 3D techniques, such as matrix shifts during experimental setup or image capture, can introduce technical artifacts that affect downstream analyses. We sought to develop an automated analytical approach for post-experimental assessment or correction of technical artifacts.

## Graphical abstract

**Figure:**
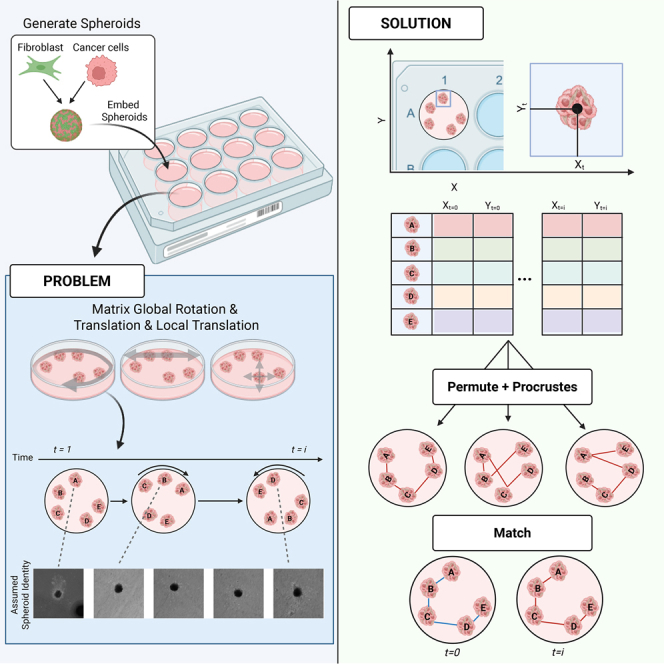

## Teaser

Cramer et al. present TRACE-QC, a method for quality control of time-course imaging in 3D cell cultures. Using iterative Procrustes analysis, it reorders and aligns spheroid positions, corrects mislabeled images, and improves data integrity. TRACE-QC enhances assay reliability and interpretability without experimental modifications or parameter tuning.

## Introduction

Three-dimensional (3D) cell culture models, like tumor spheroids, are increasingly used in cancer research because they better mimic *in vivo* tumor biology, including spatial architecture, heterogeneity, cell-cell interactions, and drug resistance, compared to 2D cultures.^1^^,^^2^^,^^3^^,^^4^^,^^5^ Longitudinal imaging (bright-field, phase-contrast, and fluorescence microscopy) allows for monitoring spheroid growth, morphology, cellular processes, and responses to experimental conditions.^1^^,^^6^^,^^7^ This enables single-cell and region of interest (ROI) analyses to observe dynamic changes and spatial reorganization, facilitating high-throughput drug screening for factors like penetration, efficacy, toxicity, and microenvironmental influences (e.g., cell-cell interactions and hypoxic regions).^1^^,^^3^^,^^8^^,^^9^^,^^10^ Consequently, longitudinal monitoring of tumor spheroids is a valuable tool for preclinical research and therapeutic evaluation.^1^^,^^11^

**Figure 1:**
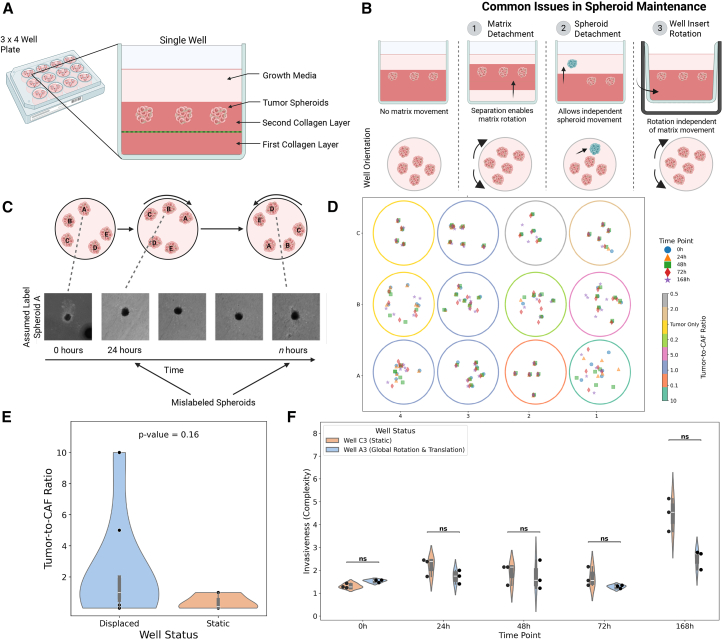
Experimental setup and collagen detachment’s effect on spheroid mislabeling (A) Schematic shows the spheroid culture system’s layered structure (collagen layers, embedded tumor spheroids, and growth media) within a well plate. (B and C) Illustrations and bright-field images depict how mislabeling and mis-recording of spheroid measurements can occur over time due to various scenarios, including well rotation. (D) Schematic of the biological dataset illustrates a 3 × 4 well plate with tumor spheroids of varying cell type ratios, showing spheroid locations at different time points. Each circle represents a well, with spheroid locations at different time points indicated by color and shape. All spheroid locations are reported using the same color and marker shape according to their associated time point. (E) A violin plot compares the tumor-to-CAF ratio in displaced vs. static wells, with no significant difference found (Kruskal-Wallis test). (F) Spheroid invasiveness (complexity) over time, categorized by well displacement status, showed no significant differences at any time point (Kruskal-Wallis test).

A major hurdle in time-series imaging of tumor spheroids is spatial displacement from experimental or technical artifacts.^1^^,^^6^ Long-term cultures, medium changes, or mechanical disruptions can shift embedded objects (e.g., if a well plate insert or collagen matrix detaches, Figures 1A and 1B). If detected, these shifts necessitate redoing experiments, wasting resources. If undetected, mislabeled images occur because spheroids are imaged at their previous locations, making it difficult to track individual spheroids accurately (Figures 1C and 1D).^10^^,^^11^^,^^12^^,^^13^ Such displacements can confound analyses, leading to misinterpretations of growth and treatment responses.^8^ The high volume of images from this high-throughput method complicates quality control for technical artifacts. Thus, computational techniques are needed to detect and quantify these errors in longitudinal imaging. Further computational corrections can mitigate wasted time and resources by recovering data from affected experiments.

Current computational approaches struggle to quantify drift and correctly label 3D cultures in longitudinal imaging for several reasons. Due to the dynamic and evolving nature of spheroids over time, matching images to the label associated with the nearest neighbor between time points is unreliable because object shape, texture, and other characteristics change, altering their appearance as they grow and invade the surrounding matrix.^7^^,^^14^^,^^15^ Low-dimensional feature representations, such as latent embedding from autoencoders, are also limited because they do not incorporate the global positional information needed to match objects across time points.^16^ Standard point set registration techniques rely on tuning a hyper-parameter, requiring ground truth data to optimize and potentially introducing biases.^17^ Traditional image registration techniques rely on fixed spatial landmarks, which are ineffective since embedded objects typically lack stable reference points, particularly when imaged as isolated objects rather than within a larger context (Figure 1C).^18^ Without images capturing the full well plate at each time point, there is no global position information for each spheroid. Existing methods for tracking the movement of objects, such as Trackmate, OrganoID, and others, require all objects being tracked to be present in the field of view at every time point.^13^^,^^19^^,^^20^^,^^21^^,^^22^^,^^23^^,^^24^ Consequently, they cannot be repurposed to resolve identity mismatches between spheroids. Additionally, without global position information, object-based landmark registration becomes infeasible. Thus, current approaches fail to provide a robust solution for detecting and correcting matrix displacement, increasing the likelihood of data misinterpretation and experimental artifacts.

To overcome these challenges, we developed an algorithm, quality control metric, and software package, TRACE-QC (temporal reassignment and correspondence evaluation with quality control) that ensures accurate spheroid matching by leveraging positional geometry across time points. Using a dataset of tumor spheroids embedded in a collagen matrix and imaged at 0, 24, 48, 72, and 168 h, we demonstrate the effectiveness of TRACE-QC in detecting and correcting matrix displacement. Our approach utilizes image metadata-derived coordinates to construct a polygon representing spheroid positions within the well. By applying an affine transformation, TRACE-QC corrects for different types of displacements including global well rotations and translations, as well as small local translations of individual spheroids. TRACE-QC enables accurately following individual spheroids’ dynamics over time, ensuring data integrity without requiring additional experimental modifications. The TRACE-QC software package provides a robust quality control step for longitudinal 3D imaging, enhancing the reliability and interpretability of spheroid-based assays in cancer research.

## Results

### Spheroid displacement was not associated with biological conditions

To investigate tumor invasiveness, we generated spheroids with varying tumor cell-to-cancer-associated fibroblast (CAF) ratios, embedded them in a collagen mixture, and replated them into pre-coated wells (Figures 1A and 1D). Each well contained four to six replicates of each cell ratio (see method details, spheroid generation) within the collagen matrix. Each spheroid was imaged at 24, 48, 72, and 168 h to monitor their growth and invasion while assessing the integrity of the surrounding matrix. We obtained a table of XYZ coordinates for all spheroids at each time point (see method details, image position acquisition). Analysis of the positional data revealed that, in 9 out of 12 wells, at least one spheroid exhibited substantial displacement during the experiment (Figure 1D). This finding highlights the prevalence of positional shifts in long-term spheroid culture experiments, necessitating a correction. Thus, we categorized the status of wells that featured spheroids with displacement as “displaced” and others “static.”

To determine whether cell type ratio influenced spheroid displacement, we assessed the relationship between tumor-to-CAF ratio and well status via the Kruskal-Wallis test (Figure 1E). The results showed no significant association between spheroid displacement and the tumor-to-CAF ratio of the spheroid, indicating that the experimental variable did not drive spheroid displacement. Next, we investigated the potential impact of well status on spheroid invasiveness (the experimental outcome measure of interest), measured with complexity—a shape descriptor calculated from each spheroid’s segmentation mask. Higher complexity values indicate greater invasiveness, as spheroids develop irregular edges and spread into the matrix.^14^ Violin plots (Figure 1F) depict the distribution of complexity values over time for spheroids composed of a 1:1 tumor-to-CAF ratio in two example wells: one where spheroid coordinates remained static and another where displacement occurred. No statistically significant differences in complexity were observed between the two groups at any time point, suggesting that matrix shifts did not alter the biological conditions of the experiment and impact the overall invasiveness of the spheroids.

Since spheroid complexity remained consistent across well status over time, we inferred that their morphological behavior—and thus invasiveness—was not affected by displacements. This stability allowed us to proceed with analyzing individual spheroid dynamics. To do so, we first needed to correct mislabeling of observations across time points by developing an algorithm to accurately pair each image with its corresponding spheroid.

### Development of TRACE-QC algorithm for matching spheroid images from longitudinal imaging and accuracy metric using simulation studies

**Figure 2:**
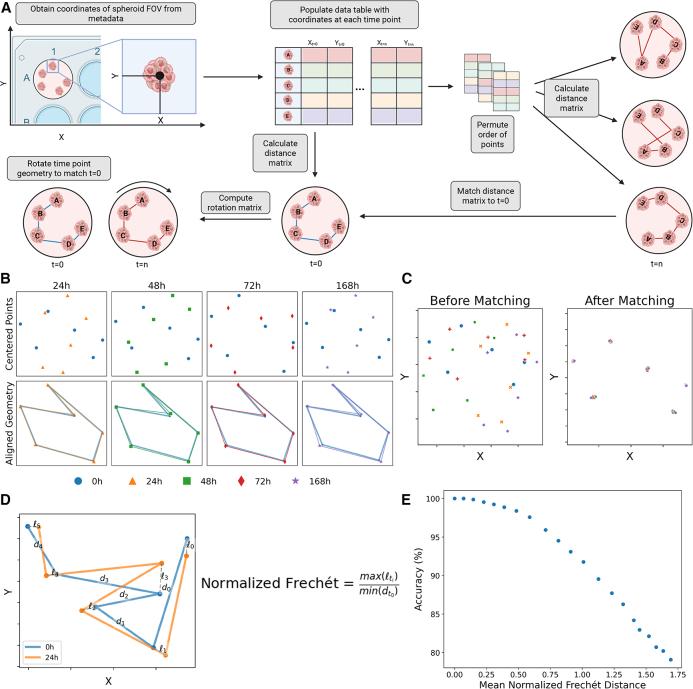
Spheroid alignment algorithm (TRACE-QC) and performance evaluation (A) TRACE-QC algorithm schematic. (B) Simulated spheroid locations (*n* = 5 spheroids) over time (0, 24, 48, 72, and 168 h) before and after alignment, with lines showing geometric transformations. Legend shared with Figure 1C. (C) Alignment results for simulated data, showing spheroid positions before and after alignment to t = 0. (D) Illustration of the normalized Fréchet metric, which quantifies curve similarity (spheroid paths) adjusted for scale by minimum nearest-neighbor distance. (E) Relationship between normalized Fréchet distance and matching algorithm accuracy from 105,000 simulated wells with increasing positional perturbations. A normalized Fréchet distance of 1.0 indicates approximately 90% accuracy.

To correct errors in image assignments caused by spheroid displacement, we developed TRACE-QC to automatically reassign mislabeled images to the correct spheroid at each time point (see method details, image matching; Figure 2A). To evaluate TRACE-QC’s performance, we first tested it on synthetic data, simulating the displacement of six spheroids under randomized spatial arrangements. Each simulation introduced random global rotations and translations and local translations (a.k.a. positional jittering), mimicking experimental disturbances. Figure 2B depicts the geometry matching process for an example simulation, with the initial unregistered spheroid positions at each time point and the resulting alignment from TRACE-QC, which rotates, translates, and stretches the connections between the simulated points to match their geometries to their configuration at the initial time point. As shown in Figure 2C, TRACE-QC successfully matched spheroids at each time point, restoring their spatial relationships to the initial baseline configuration.

To quantitatively assess the performance of TRACE-QC, we adapted the Fréchet distance, which measures the smallest maximum distance necessary to connect two paths of points in space while accounting for the ordering of the points.^25^ To allow comparisons across different spatial scales, we normalized the Frechét distance by dividing it by the minimum initial distance between any two spheroids (Figure 2D). We chose to normalize by the smallest distance because it represents a conservative estimate for the threshold at which a spheroid’s nearest neighbors would change in the squared Euclidean distance matrix, and subsequently compromise the permutation strategy in the first step of our algorithm.

To evaluate TRACE-QC’s robustness, we simulated displacement of a single spheroid at varying distances from its original position, ranging from 0% to 200% of the minimum nearest-neighbor distance, increasing in 10% increments. Each perturbation level was tested across 100 independent simulations, and the algorithm was applied to 50 randomly generated initial spheroid configurations. TRACE-QC’s accuracy was determined based on the percentage of correctly matched spheroids for each simulation. For each perturbation level, the average accuracy was plotted against the average normalized Frechét distance to establish a relationship between our performance metric and algorithm accuracy (Figure 2E). The results showed that a normalized Frechét distance of 1.0 corresponded to approximately 90% accuracy, establishing a threshold for extreme perturbations. Notably, this metric can also be used for automated decision-making, eliminating the need for manual visual evaluation of the geometry graph (Figure 2B). The TRACE-QC algorithm effectively corrected for global well rotations and translations, along with random local spheroid displacements (translations) within the range of typical experimental shifts.

We benchmarked TRACE-QC against alternative methods for tracking the movement of objects via centroid positions. We compare TRACE-QC to the Python implementations of the linear assignment problem algorithm (the default tracker in the Trackmate Fiji plugin), as well as the Crocker-Grier algorithm integrated by deep learning trackers.^19^^,^^26^^,^^27^^,^^28^ We simulated the displacement of six spheroids undergoing random global rotations, global translations, and local displacements and found that TRACE-QC consistently outperformed the alternatives in this spheroid tracking and matching task (Figures S1 and S2A–S2D). We further evaluated TRACE-QC against sorting the correspondence space (SCS),^29^ a parameter-free point set registration technique, by simulating the displacement of increasing numbers of spheroids with randomized global rotations, global translations, and local displacements. The results demonstrated near-equivalent performance, with TRACE-QC exhibiting modest but consistent improvements (Figure S2E).

### Matching algorithm identifies and corrects displacement errors in experimental data

**Figure 3:**
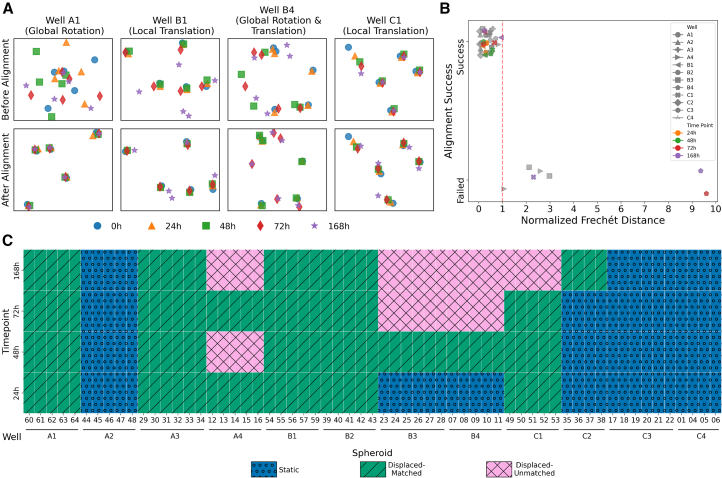
TRACE-QC performance on biological spheroid data (A) Examples of successful, partial, and failed matching and single-spheroid local translation. Panels display spheroid positions pre-/post-TRACE-QC. (B) Normalized Frechét distance vs. matching success/failure, with highlighted examples. Dashed line at 1.0 indicates alignment success cutoff. (C) Heatmap summarizing spheroid status after TRACE-QC: static, matched, or unmatched, relative to time point.

After validating the robustness of our algorithm on simulated data, we applied TRACE-QC to each well in the plate from our dataset of tumor spheroids with varying tumor-to-CAF ratios, imaged over 168 h (Figures 1D, S3, and S4). Representative examples in Figure 3A of wells from the well plate illustrate TRACE-QC’s performance across a range of scenarios: successful matching in wells with varying displacement level (Well A1); partial success, where certain time points were accurately matched while others were poorly matched (Well B1); a case where matching failed due to extreme displacement or confounding factors (Well B4); and a case where a single spheroid’s coordinates shifted over time (Well C1). Thus, TRACE-QC successfully paired spheroids with their correctly associated images across a variety of scenarios. An example of manual visual inspection for a displaced well is shown in Figures S5A and S5B.

To quantify the success of matching, we calculated the normalized Frechét distance for each well at each time point and compared it between successfully and unsuccessfully matched wells (Figure 3B). Wells with normalized Frechét distances below 1.0 consistently exhibited successful matching, supporting simulation findings that extreme positional shifts reduce TRACE-QC’s accuracy (Figure 2E). Thus, our normalized Frechét distance can serve as a quantitative quality control measure of spheroid displacement over the course of experiments. Additionally, we observed that smaller nearest-neighbor distances at the initial time point (t = 0) can cause increased normalized Frechét distances, suggesting that spheroids should not be embedded too proximally to each other to facilitate accurate matching (Figures 3A and 3B, Well B4). The net change in normalized Frechét distance before and after correction with TRACE-QC is shown in Figures S5C and S5D.

To establish a comprehensive assessment of TRACE-QC’s ability to correctly label spheroids and recover the dynamics of individual spheroids across the experimental dataset, we calculated the matching success for each spheroid at every time point. We found that nine of the twelve wells demonstrated spheroid coordinate displacement during at least one time point, leading to uncertainty in 66% of the total measurements collected during the experiment. TRACE-QC successfully recovered or validated 77% of potentially mislabeled spheroid measurements (Figure 3C), demonstrating its ability to deconvolute confounded data and improve experimental reliability.

## Discussion

This study addresses a critical challenge in longitudinal imaging of tumor spheroids—mislabeling images of spheroids due to local shifts in individual spheroids or by global rotation or translation of well matrices or well plate inserts over time. Alternative experimental modification could be considered to ensure that images of tumor spheroids will be correctly labeled. For instance, incorporating well plate inserts with grids etched beneath the matrix or embedding fiducial markers could provide fixed reference points for matching or alignment.^30^ Fabrication of microwells embedded in the matrix will help with isolating individual spheroids and may limit local displacements but will not prevent global translations and rotations of the matrix or a well plate insert. Alternatively, acquiring large scan images of the entire well plate at each time point, in addition to individual fields of view, would allow for *post hoc* reconstruction of spheroid trajectories even in the presence of matrix displacement. However, these alternatives introduce additional complexity or cost, such as new equipment or data storage requirements, to the experimental workflow.

TRACE-QC provides a robust, flexible, and efficient solution that ensures accuracy when following the dynamic behaviors of tumor spheroids over time. By calculating the normalized Frechét distance after alignment, researchers can determine the degree of change experienced by their spheroids over the course of an experiment and evaluate algorithm success. Another key strength of this approach is its adaptability to existing experimental workflows. As it relies solely on field-of-view coordinates extracted from imaging metadata, TRACE-QC is broadly applicable to different types of assays that use longitudinal imaging of 3D cultures such as organoids embedded in extracellular matrix. The implications of this algorithm therefore extend beyond the specific context of spheroid matching presented in this study. By relying solely on image coordinates, the algorithm may be applied to data generated by a wide range of experimental protocols and imaging platforms and be used prospectively to ensure data integrity in new studies or retrospectively to analyze existing datasets.

### Limitations of the study

The biological dataset with which we validate TRACE-QC is small. This may limit inference of potential biological findings in this study, but it does not detract from the demonstration of the algorithm itself. The second limitation is intrinsic to TRACE-QC as performance depends on the initial spatial arrangement of objects. If objects are positioned in a perfectly symmetrical polygonal configuration (e.g., a square or regular hexagon), rotational symmetry can lead to ambiguities in alignment, making it difficult to correctly permute objects across time points. In addition, the permutation step of our algorithm requires calculating pairwise differences between all point locations. This step can be computationally demanding, with SCS representing a more efficient alternative. Consequently, TRACE-QC assumes that there is the same number of spheroids at each time step. Finally, matrix degradation or fragmentation over time can introduce inconsistencies, further challenging the ability to maintain object correspondence. Importantly, TRACE-QC failure may occur in cases of extreme geometric misalignment during the second step of the algorithm. It can also happen due to changes in the total number of spheroids at each time step or because of matrix degradation. Such failures may serve as a useful quality control step to indicate fusion events between spheroids, dissolution of spheroids, or potential structural instability within the matrix, thereby highlighting issues within the experimental set up.

Ultimately, TRACE-QC and the normalized Frechét present a systematic and unbiased technique to ensure that the observed phenotypes of tumor spheroids embedded in collagen matrices are not related to an experimental artifact. This is critical for studies looking at the dynamic behaviors or spatial arrangements of single cells within regions of interest of a matrix-embedded object.^8^^,^^14^^,^^31^ Furthermore, provided measurements of biological interest remain consistent across displaced and static conditions, TRACE-QC may maximize the value of existing experiments by recovering previously unusable data affected by matrix displacement. The core principles of the TRACE-QC algorithm—permutation optimization combined with Procrustes alignment—are dimension agnostic and can be readily adapted to other applications involving spatial misalignment, such as organoid invasion analysis, correctly aligning images with respect to their Z coordinates, or aligning tissue architecture landmarks across adjacent sections.^32^^,^^33^

## Resource availability

### Lead contact

Requests for further information and resources should be directed to and will be fulfilled by the lead contact, Dr. Young Hwan Chang (chanyo@ohsu.edu).

### Materials availability

This study did not generate new unique reagents.

### Data and code availability

• All data generated in this study are available within the article and available on Zenodo: https://doi.org/10.5281/zenodo.17196124.
• The software code together with detailed instructions can be found on Zenodo: https://doi.org/10.5281/zenodo.16590562, and GitHub: https://github.com/emcramer/TRACEQC.
• Any additional information needed to reanalyze the data reported in this paper is available from the lead author by request.

## Acknowledgments

We thank Dr. David Tuveson, Dr. Dennis Plenker, Dr. Amber Habowski, and Hardik Patel for kindly sharing the hT231 cell line and Dr. Elizabeth Jaffee for her mentorship. We acknowledge Genevieve Stein-O’Brien and Paul Macklin and thank them for their feedback. This work was supported by the 10.13039/100016460Jayne Koskinas Ted Giovanis Foundation for Health and Policy grant awarded to L.M.H. E.J.F. was supported by NIH/NCI U24CA284156; A.W. and V.W. were supported by the T32CA153952; E.M.C. was supported by the NIH/NCI 1T32CA254888-01 and by the NIH/NIGMS 5T32GM141938-04. J.W.Z. and T.L.-V. report funding for the spheroid and CAF experiments from the Charles and Margaret Levin Family Foundation and the Dana & Albert R. Broccoli Charitable Foundation. The research reported in this publication used computational infrastructure supported by the Office of Research Infrastructure Programs, Office of the Director, of the 10.13039/100000002National Institutes of Health under award number S10OD034224. The content is solely the responsibility of the authors and does not necessarily represent the official views of the National Institutes of Health. Figures 1 and 2 were made with BioRender.

## Author contributions

E.M.C.: conceptualization, data curation, formal analysis, methodology, project administration, software, validation, visualization, writing – original draft, and writing – review and editing. T.L.-V.: conceptualization, data curation, investigation, methodology, visualization, writing – original draft, and writing – review and editing. J.J.: conceptualization, data curation, writing – original draft, and writing – review and editing. V.W.: investigation. D.R.B.: conceptualization and supervision. A.W.: funding acquisition and supervision. R.B.: funding acquisition and supervision. J.W.Z.: funding acquisition, supervision, and writing – review and editing. E.J.F.: funding acquisition, supervision, project administration, and writing – review and editing. L.M.H.: funding acquisition, supervision, project administration, and writing – review and editing. Y.H.C.: conceptualization, methodology, funding acquisition, supervision, project administration, and writing – review and editing.

## Declaration of interests

E.J.F. was on the scientific advisory board of Resistance Bio/Viosera Therapeutics; was a paid consultant for Mestag Therapeutics and Merck; and received grants from Roche/Genentech, Abbvie Inc, National Foundation for Cancer Research, and Break Through Cancer outside the scope of this work. J.W.Z. reports grant funding support (to Johns Hopkins) and travel from Roche/Genentech and funding from Break Through Cancer outside the submitted work and honoraria from Sermo and ZoomRx.

## STAR★Methods

### Key resources table

**Table:** 

REAGENT or RESOURCE	SOURCE	IDENTIFIER
**Chemicals, peptides, and recombinant proteins**		

RPMI-1640	Thermo Fisher Scientific	Cat# 11-875-085
FBS	GeminiBio	Cat# 100-106
Amphotericin B	Sigma	Cat# A2942
Penicillin-Streptomycin	Gibco	Cat# 5140122
UltraPure Agarose	Invitrogen	Cat# 16500100
Rat Tail Collagen I	Gibco	Cat# A10483-01
1x PBS	Corning	Cat# 21-040-CV
EMEM Media	Quality Biological	Cat# 112-018-101
L-glutamine	Corning	Cat# 25-005-CI
NaHCO_3_	Quality Biological	Cat# 118-085-721
DMSO (Dimethyl Sulfoxide)	Sigma	Cat# 472301
Trypsin-EDTA (0.25%)	Gibco	Cat# 25200056
MEM Nonessential Amino Acids, 100x	Corning	Cat# 25-025-CI
Sodium Pyruvate	Corning	Cat# 25-000-CI
Humulin R (Regular Human Insulin)	Eli Lilly	Cat# 00002-8215-01

**Critical commercial assays**		

MycoStrips	Invivogen	Cat# rep-mys-100
CytoPainter Cell Proliferation Staining Reagent (Deep Red Fluorescence)	Abcam	Cat# ab176736
CytoPainter Cell Proliferation Staining Reagent (Green Fluorescence)	Abcam	Cat# ab176735

**Deposited data**		

AlignSpheroid dataset	This paper	Zenodo: https://doi.org/10.5281/zenodo.17196124

**Experimental models: Cell lines**		

Panc10.05	Jaffee et al.^34^ available through ATCC	Cat# CRL-2574
hT231	Dr. David Tuveson, Dr. Dennis Plenker, Dr. Amber Habowski, and Hardik Patel	N/A

**Software and algorithms**		

TRACE-QC	This paper	GitHub: https://github.com/emcramer/TRACEQC Zenodo: https://doi.org/10.5281/zenodo.16590562
SciPy	Virtanen et al., 2020^35^	RRID: SCR_008058; https://scipy.org/
NumPy	Harris et al., 2020^36^	RRID: SCR_008633; https://numpy.org/
Python	Python.org	RRID: SCR_008394; https://python.org
Napari	Chiu et al., 2022^37^	RRID: SCR_022765; https://napari.org/stable/
Matplotlib	Hunter et al., 2007^38^	RRID: SCR_008624; https://matplotlib.org/
Seaborn	Waskom and the seaborn development team^39^	RRID: SCR_018132; https://seaborn.pydata.org/
Pandas	McKinney and the pandas development team^40^	RRID: SCR_018214; https://pandas.pydata.org/
Scikit-image	Van der Walt and the scikit-image development team^41^	RRID: SCR_021142; https://scikit-image.org/
Segment Anything for Microscopy	Archit et al., 2025^42^	https://github.com/computational-cell-analytics/micro-sam

**Other**		

96 Well Polypropylene Plates	Celltreat	Cat# 229576
24-well SensoPlate™	Greiner Bio-One	Cat# 662892
6-well Cell culture plates	GenClone	Cat# 25-105
PES Vacuum Filtration Systems, 500mL, 0.22 μm filter	GenClone	Cat# 25-227
Cell culture flasks	GenClone	Cat# 25-209

### Experimental model and study participant details

#### Cell lines

The human CAF cell line hT231 was generated by digesting a resected pancreatic tissue sample following standard organoid line establishment protocols.^43^ The undigested fibrous tissue was then placed into cell culture plates with DMEM supplemented with 10% fetal bovine serum (FBS, Thermo Fisher Scientific) and penicillin-streptomycin (Gibco, 5140122). The 2D cells that proliferated were then immortalized by viral transduction with the SV40 LT-pLVX-SV40 LT-IRES-tdTomato plasmid, as previously described.^44^ The hT231 cell line and a pancreatic tumor cell line Panc10.05 and were cultured in RPMI 1640 Medium with L-Glutamine (RPMI, Thermo Fisher Scientific, 11-875-085) with 10% FBS, 0.1% Amphotericin B (Sigma, A2942), and 1% penicillin-streptomycin.^34^ Both cell types were Mycoplasma tested using Invivogen MycoStrips (rep-mys-100). The sex and gender of Panc10.05 are reported by ATCC. The HT-231 cell line lacks a fingerprint for authentication due to patient anonymization. For this experimental context, these variables are not considered relevant to measured biological endpoints.

All cell cultures were maintained at 37°C with 5% CO_2_ and passaged upon reaching 80–90% confluence. For passaging, cells were washed with PBS, treated with trypsin-EDTA, and incubated at 37°C for 5 min. An equal volume of culture medium was then added to quench trypsin activity. The cell suspension was centrifuged, resuspended in fresh medium, and seeded at a 1:10 ratio into new flasks.

### Method details

#### Cell staining

To differentiate cells, CytoPainter Cell Proliferation Staining Reagents (Abcam) were used to stain each cell type. Panc10.05 was labeled for green fluorescence (ab176735), and HT231 was labeled for red fluorescence (ab176736), following the manufacturer’s instructions.

#### Spheroid generation

A 96-well plate (Celltreat, 229576) was treated with 50 μL of 1.5% Invitrogen UltraPure Agarose (#16500100) and allowed to solidify for 1 h at room temperature. Panc 10.05 cells were mixed with hT231 cells at 1:0, 1:1, 2:1, 5:1, 10:1, 1:2, 1:5 and 1:10, tumor cell-to-CAF ratios. For each ratio, 4–6 technical replicates of 10,000 cells/well were seeded in a final volume of 200 μL/well of culturing media and onto the 1.5% agar-treated plate. Plates were maintained at 37°C 5% CO_2_ for 4 days until spheroid formation was microscopically observed using the Nikon ECLIPSE Ti2 inverted microscope.

The spheroids were resuspended in 300 μL of Collagen Mix, which consists of 37% Rat Tail Collagen 1, 37% PBS (Corning, 21-040-CV), 10% FBS, 11.3% EMEM Media (Quality Biological, 112-018-101), 0.8% L-glutamine, and 5% NaHCO3 (Quality Biological, 118-085-721).^7^ Each spheroid ratio was then replated into a well of a 24-well SensoPlate (Greiner Bio-One, 662892), which had been pre-coated with 300 μL of the same Collagen Mix. Each well contained six spheroid replicates with identical ratios of Panc 10.05 and hT231 cells distributed within the collagen matrix.

#### Image position acquisition

To visualize spheroid invasion, imaging was carried out with a Nikon ECLIPSE Ti2 inverted microscope using 1x, 10x and 20x objectives. Spheroids were imaged at 0, 24, 48, 72, and 168 h after embedding. For each spheroid, five z stack images were taken across a 60 μm range using both brightfield and fluorescent imaging, with fluorescence captured using 647 nm and 488 nm lasers. The cell culture plates were placed on a live cell stage and maintained at 37°C with 5% CO_2_ during imaging. At the initial time point (t = 0), a large scan image of the entire well plate was captured using a 1x objective lens with the 25 mm field of view. From the compiled image, spheroid positions were manually selected and saved as an XY Multipoint file. For each subsequent time point, the multipoint file from the initial time point was loaded, each spheroid was manually centered using the 10x objective, and the Z-plane was refocused while keeping the PFS (Perfect Focus System) active. This ensured that all spheroid locations were imaged sequentially, reducing the need for manual adjustments. In cases where spheroids shifted significantly and fell outside the predefined FOV, the team manually scanned using the 4x objective to locate the nearest spheroid and update the XY Multipoint positions prior to acquisition. This approach ensured that all spheroids remained within the FOV across time points. If two spheroids appeared within the same FOV, the team treated each spheroid as a separate acquisition image and centered each independently in the XY Multipoint file.

The collected spheroid images were then saved in a Nikon multipoint file (.nd2) for that time point. For each time point’s ND2 file, the X and Y coordinate tables were extracted from the metadata and exported. Meanwhile, the multipoint file was split into single-frame files and stored in a separate directory for downstream analysis.

#### Image matching

We developed a custom algorithm, which we have named “TRACE-QC” (Temporal Reassignment and Correspondence Evaluation with Quality Control), to correct for potential displacement of tumor spheroids within wells of the well plate by matching the coordinates of images at later time points to the coordinates of the image taken at the first time point. Using well boundary coordinates and the X and Y locations of individual spheroid images from the metadata of the multipoint files (see method details section on image position acquisition), spheroids were assigned to their respective wells at each time point (t = 0, 24, 48, 72, and 168 h). The first step of the TRACE-QC algorithm establishes a reference configuration of the spheroids at the initial time point (t = 0). For each subsequent time point, spheroid coordinates were centered by subtracting the mean X and Y positions. Pairwise squared Euclidean distances between spheroids were calculated for both the reference and current time point configurations. The optimal ordering of spheroids at the current time point was identified by a permutation-based optimization strategy as follows. This strategy iteratively permuted the order of spheroids, calculated the pairwise squared Euclidean distance matrix for each permutation, and selected the permutation that minimized the induced L2 norm of the difference between the current and reference distance matrices (Figure 2A). The induced L2 norm was chosen for this application because it would optimize for the smallest of pairwise distances between each set of coordinates without assumptions about the order of the points.^45^

The second step of the algorithm is an alignment process that applies Procrustes analysis to determine the optimal rotation and translation to the optimal permutation of the spheroids identified during the first step. Procrustes analysis is a statistical method to compare shapes by minimizing the sum of squared distances between corresponding points and transforming them into a state of maximal superimposition.^46^ This technique finds the optimal rotation, translation, and scaling to align two or more sets of data points. Thus, it is well suited to align the reordered current time point configuration to the reference configuration taken from the first timepoint. In our implementation, the coordinates of the reference configuration and the optimal permuted configuration are first centered by subtracting each configuration’s centroid. The singular value decomposition (SVD) of the centered coordinates is used to calculate a rotation matrix and translation vector. Next, the rotation matrix and translation vector are applied to the centered coordinates of the optimal permuted configuration to align the permuted configuration to the reference. The optimal permutation of the points is then used to re-order the raw coordinates to correspond to their aligned counterparts and assign correct labels to each image.

The detailed steps of the algorithm, including the Procrustes analysis and permutation optimization, are outlined as pseudocode in Algorithm 1. Specifically, let *A* be an n x 2 matrix representing the centered reference configuration and *B* be an n x 2 matrix representing the centered, reordered current time point configuration. The algorithm proceeds by calculating the SVD of *B*^*T*^*A* and uses the resulting matrices to compute the optimal rotation and translation. This procedure robustly aligned spheroid locations across time, correcting for potential displacements and rotations of the spheroids within the well.

Algorithm 1 **Spheroid pattern matching**

### Quantification and statistical analysis

#### Quantifying invasiveness

Each image for each spheroid was analyzed using Python version 3.10. The microSAM and napari packages were used to generate initial segmentation masks for each spheroid, and masks were post-processed to fill gaps and remove noise using the scikit-image and scipy packages.^35^^,^^37^^,^^41^^,^^42^^,^^47^ The complexity of the contour in each mask was analyzed to quantify the invasiveness of each spheroid at each point in time (t = 0, 24, 48, 72, and 168 h).

#### Statistical analysis

After matching and image label correction, the trajectories of each spheroid’s invasiveness between the rotated and adhered conditions were compared via a non-parametric Kruskal-Wallis test using Python 3.10 and the scipy package^35^ significance levels are denoted by: n.s., not significant; ∗*p* < 0.05; ∗∗*p* < 0.01; ∗∗∗*p* < 0.001. Details of statistical tests and sample sizes can be found in the results text and in figure legends.

## Supplemental information

Document S1. Figures S1–S5

Document S2. Article plus supplemental information
