# Supplementary material for: Temporal reassignment and correspondence evaluation with quality control for time-course imaging of 3D cell culture
Source: Cell Rep Methods. 2025 Nov 18;5(12):101237. doi: 10.1016/j.crmeth.2025.101237 (PMC12859480; doi:10.1016/j.crmeth.2025.101237)
Supplement: Document S1. Figures S1–S5 [file mmc1.pdf]

Cell Reports Methods, Volume *5*

Supplemental information

Temporal reassignment and correspondence evaluation with quality control for time-course imaging of 3D cell culture

Eric M. Cramer, Tamara Lopez-Vidal, Jeanette Johnson, Vania Wang, Daniel R. Bergman, Ashani Weeraratna, Richard Burkhart, Elana J. Fertig, Jacquelyn W. Zimmerman, Laura M. Heiser, and Young Hwan Chang

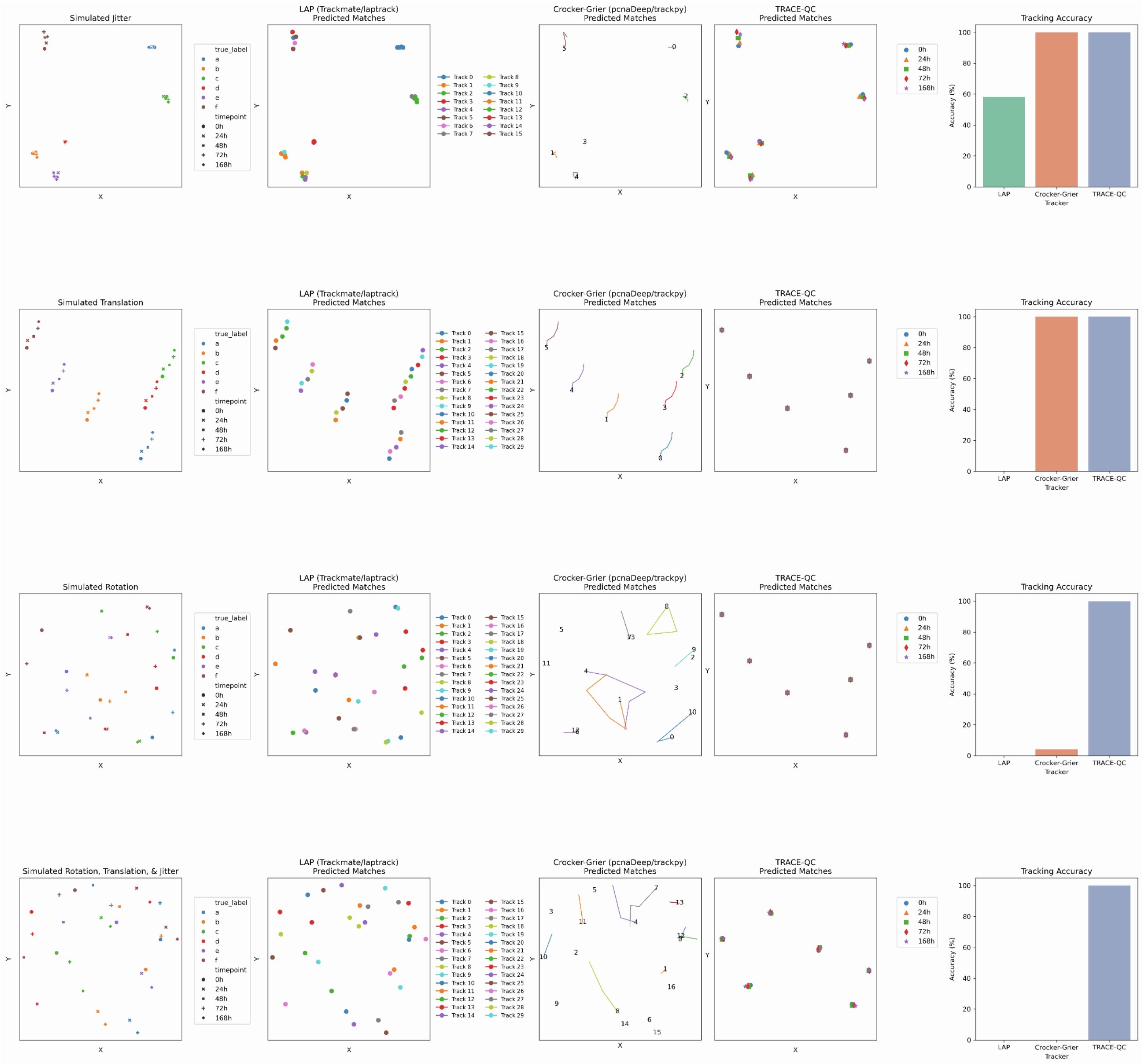

Supplemental Figure 1: Tracking and alignment algorithm accuracy comparison, related to Figure 2. The performance of different algorithms on a single simulation of each category. The first column shows the simulated spheroid data set (or well in a well plate). The second column depicts the performance of the LAP tracker implemented in the python laptrack library (and used by Trackmate version 7). The third column shows the tracking results from the Crocker-Grier algorithm. The fourth column shows the results from our TRACE-QC algorithm. The fifth column depicts each algorithm's accuracy based on the ground truth labels. Each row corresponds to a different simulated spatial perturbation (from top to bottom: jittering, translation, rotation, and all three combined).

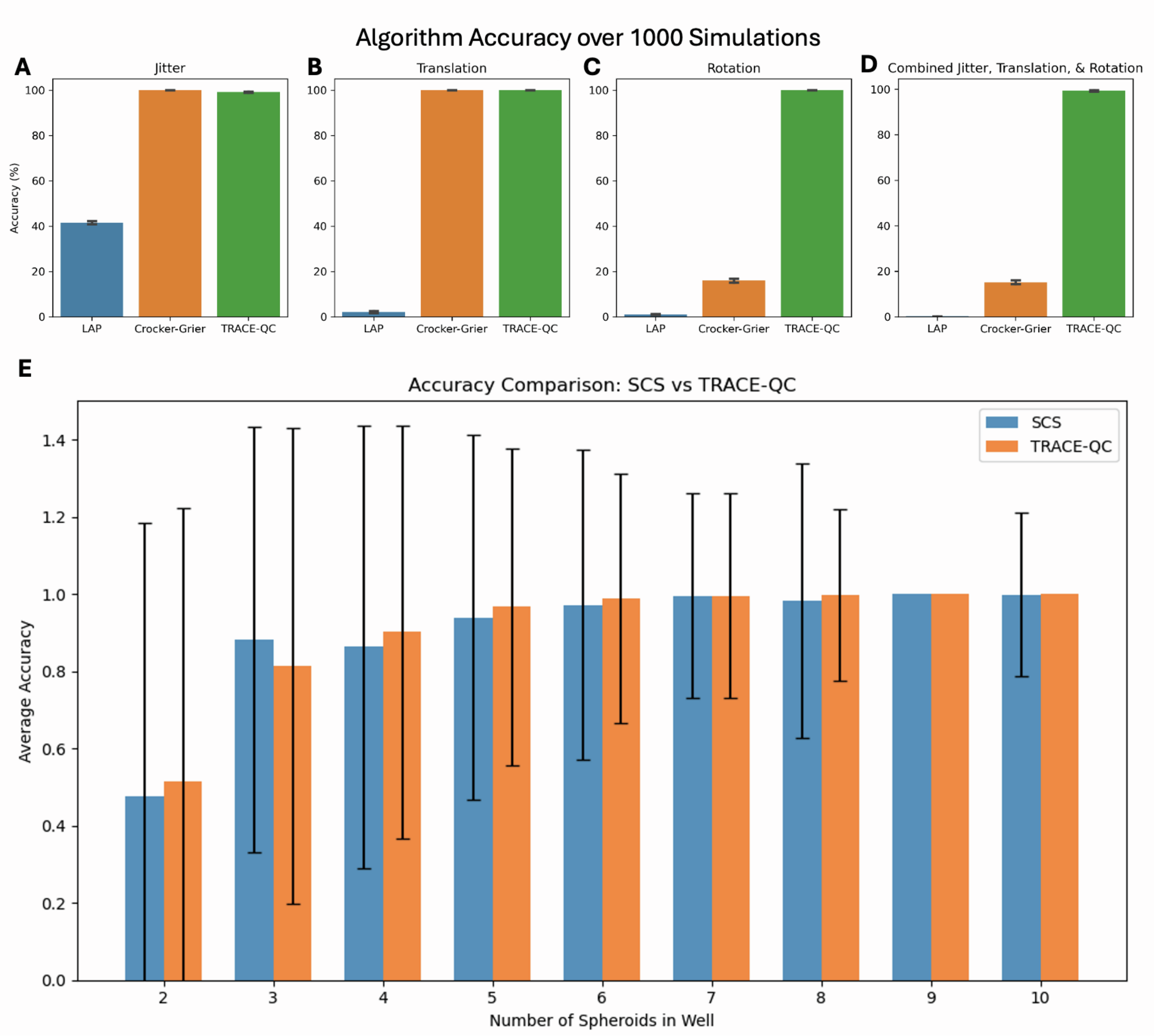

Supplemental Figure 2: TRACE-QC comparison with tracking and SCS algorithms, related to Figure 2. A-D) The four spatial displacement types (jittering - A, translation - B, rotation - C, and all three combined - D) were simulated 1000 times and each algorithm's accuracy was calculated for every simulation. Data are represented as mean ± SEM. **E)** Performance of SCS and TRACE-QC on simulated wells experiencing combinations of global rotation, global translation, and local translation (individual spheroid jittering). Data are represented as mean ± SD.

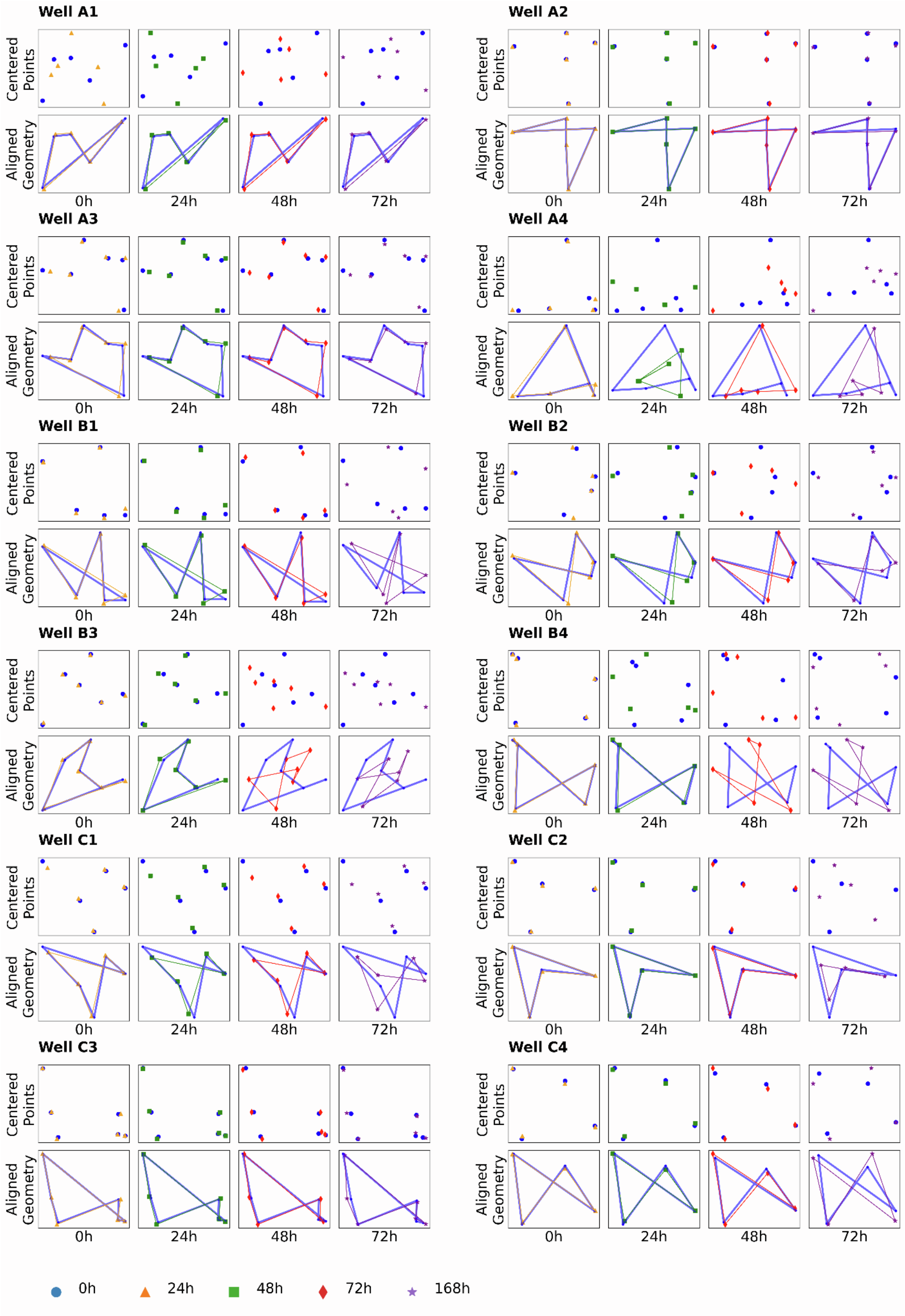

Supplemental Figure 3: Polygon visualization of correction with TRACE-QC, related to Figure 3. Application of TRACE-QC to the biological data set. The top row of each sub-panel depicts the X and Y

coordinates of the spheroids after being centered, and the bottom row depicts the geometry of the points after Procrustes analysis.

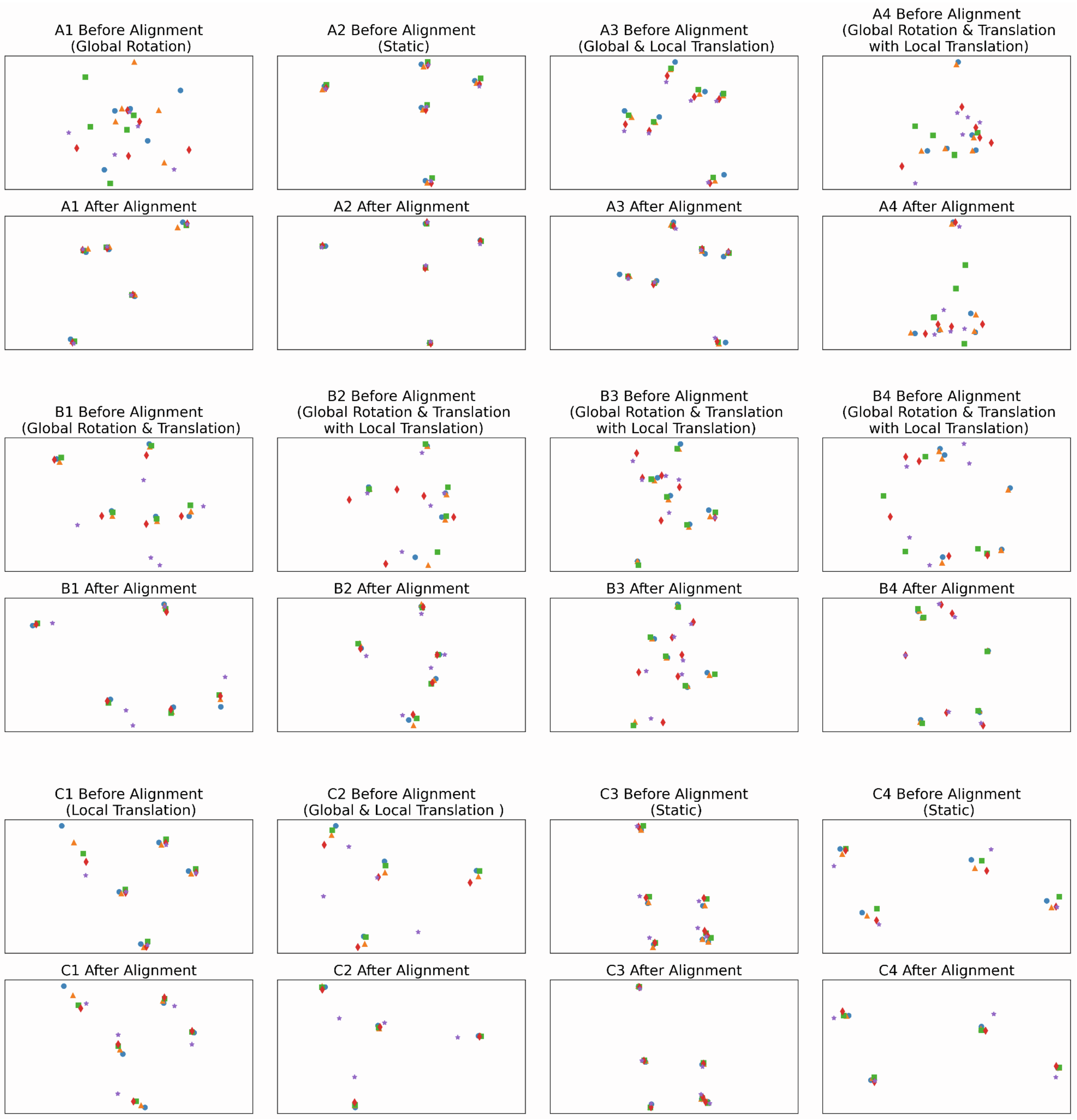

Supplemental Figure 4: Spheroid positions before and after application with TRACE-QC, related to Figure 3. The positions of spheroids before and after applying the TRACE-QC.

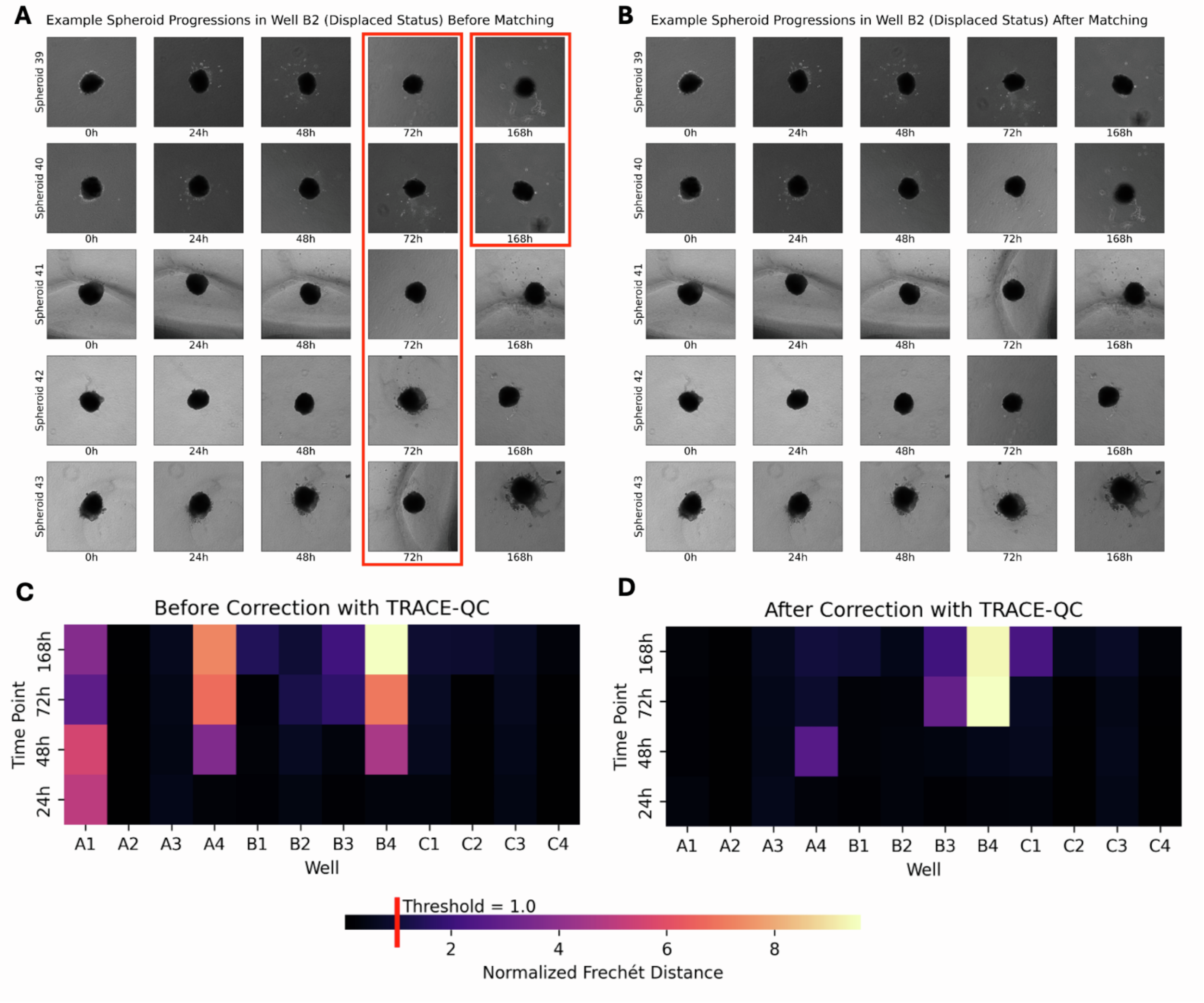

Supplemental Figure 5: Example of spheroid mismatch and correction with normalized Frechét distances before and after correction, related to Figure 3. A,B) Example application of TRACE-QC to a well with displaced spheroids in red before (Left) and after matching (Right). **C,D)** Comparison of the Normalized Frechét Distances for each of the wells in the well plate before (C) and after (D) correction with TRACE-QC.
